# Supplementary material for: Ectopic Expression of OsJAZs Alters Plant Defense and Development
Source: Int J Mol Sci. 2022 Apr 21;23(9):4581. doi: 10.3390/ijms23094581 (PMC9103030; doi:10.3390/ijms23094581)
Supplement: Supplementary file 1 [file ijms-23-04581-s001.zip › Supplementary Figure S1-S6.pdf]

Supplement figures

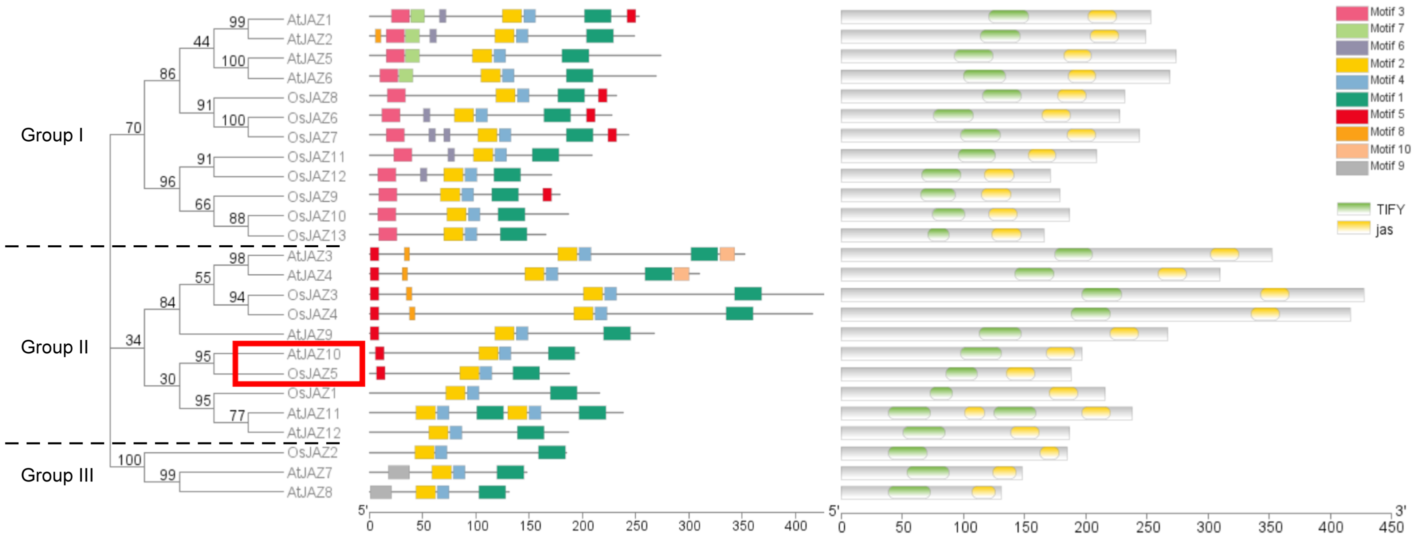

Figure S1. Phylogenetic analysis of JAZ family members. The tree is based on alignments of the full-length protein sequences and was generated using the MEGA 7 program by the neighbor-joining method. Numbers at the nodes indicate bootstrap support values (1000 replicates).

We predicted the motif of JAZ proteins using MEME and visualized them on the right side of the phylogenetic tree. The rightmost part of Figure S1 shows the positions of the TIFY domain and the jas domain in the protein. The X-axis in the picture represents the length of the protein.

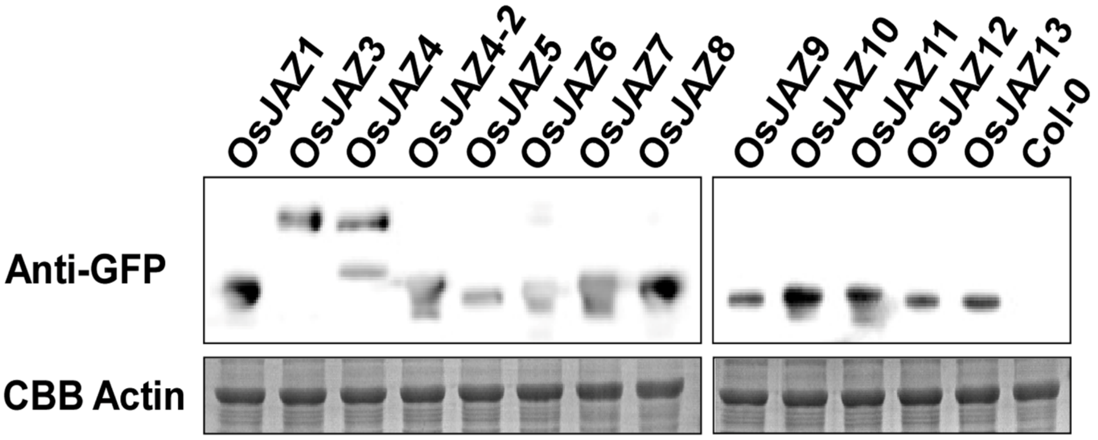

Figure S2. Exepression of OsJAZ proteins in transgenic *Arabidopsis*. 50 µg of total proteins from rosette leaves of 28 days old Arabidopsis plants were loaded into each lane for immunoblot analysis with an anti-GFP antibody. Coomassie brilliant blue (CBB) was used to stain the blotted membrane to show equal loading of proteins.

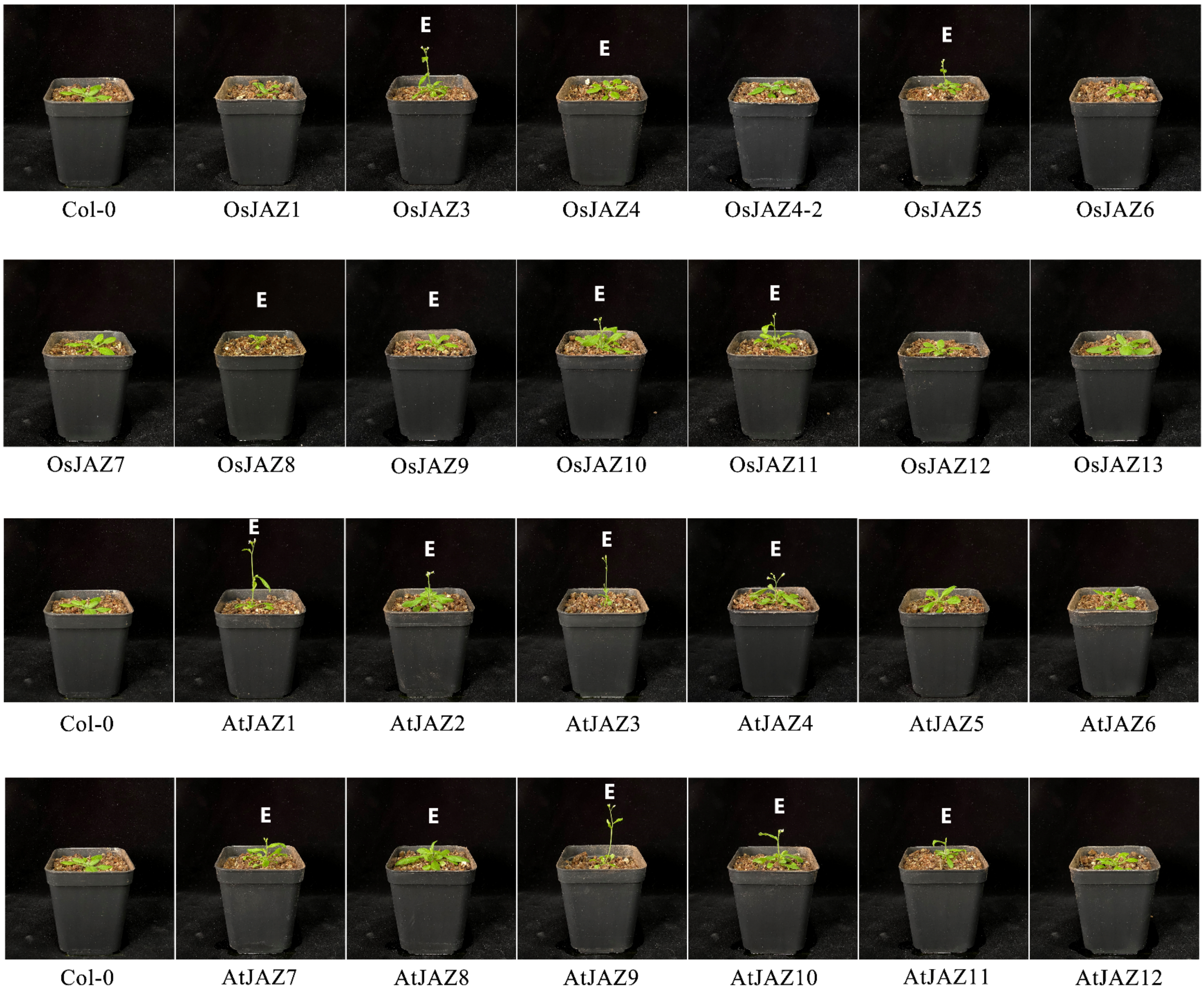

Figure S3. Several *JAZ* OE plants showed an early-flowering phenotype. The images were taken after *Arabidopsis thaliana* for 30 days. The *Arabidopsis thaliana* plants were grown in a growth chamber at 22℃ under 16 h of light (100 μE·m^{-2}·s^{-1}) and 8 h of darkness. Early flowering lines are marked with an "E".

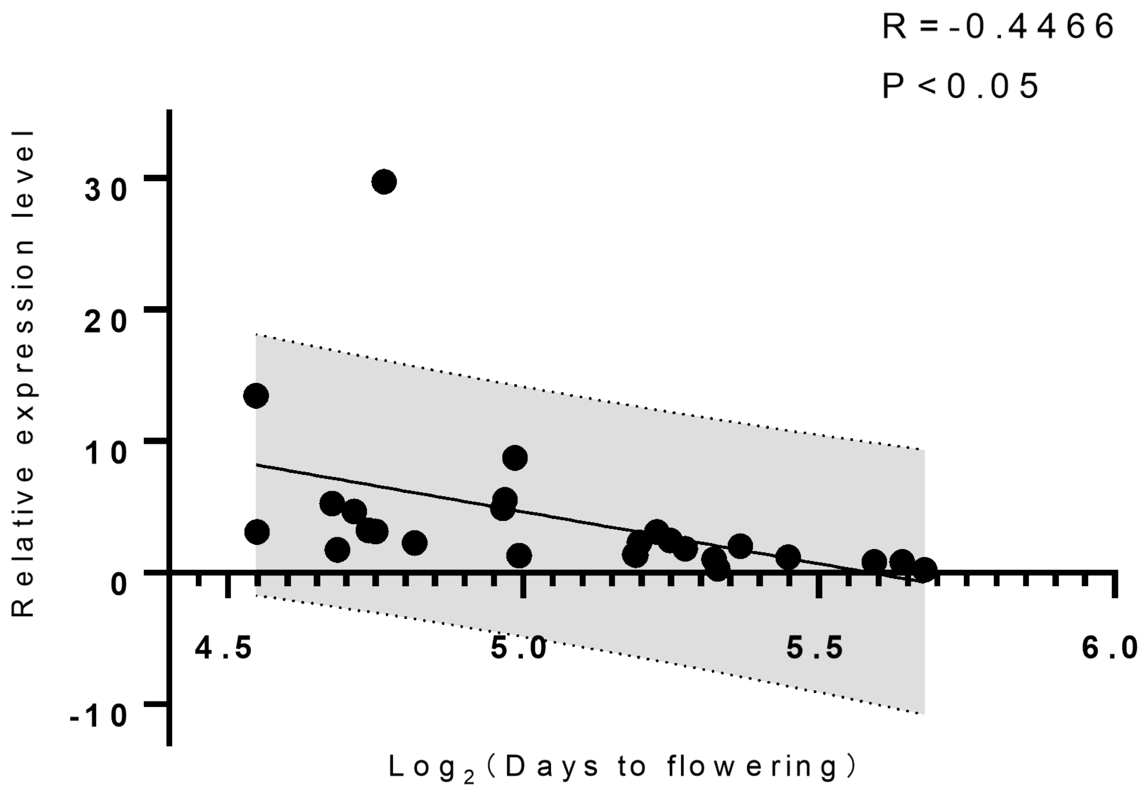

Figure S4. Correlation analysis showing that the expression level of *FT* is negatively correlated with the flowering.

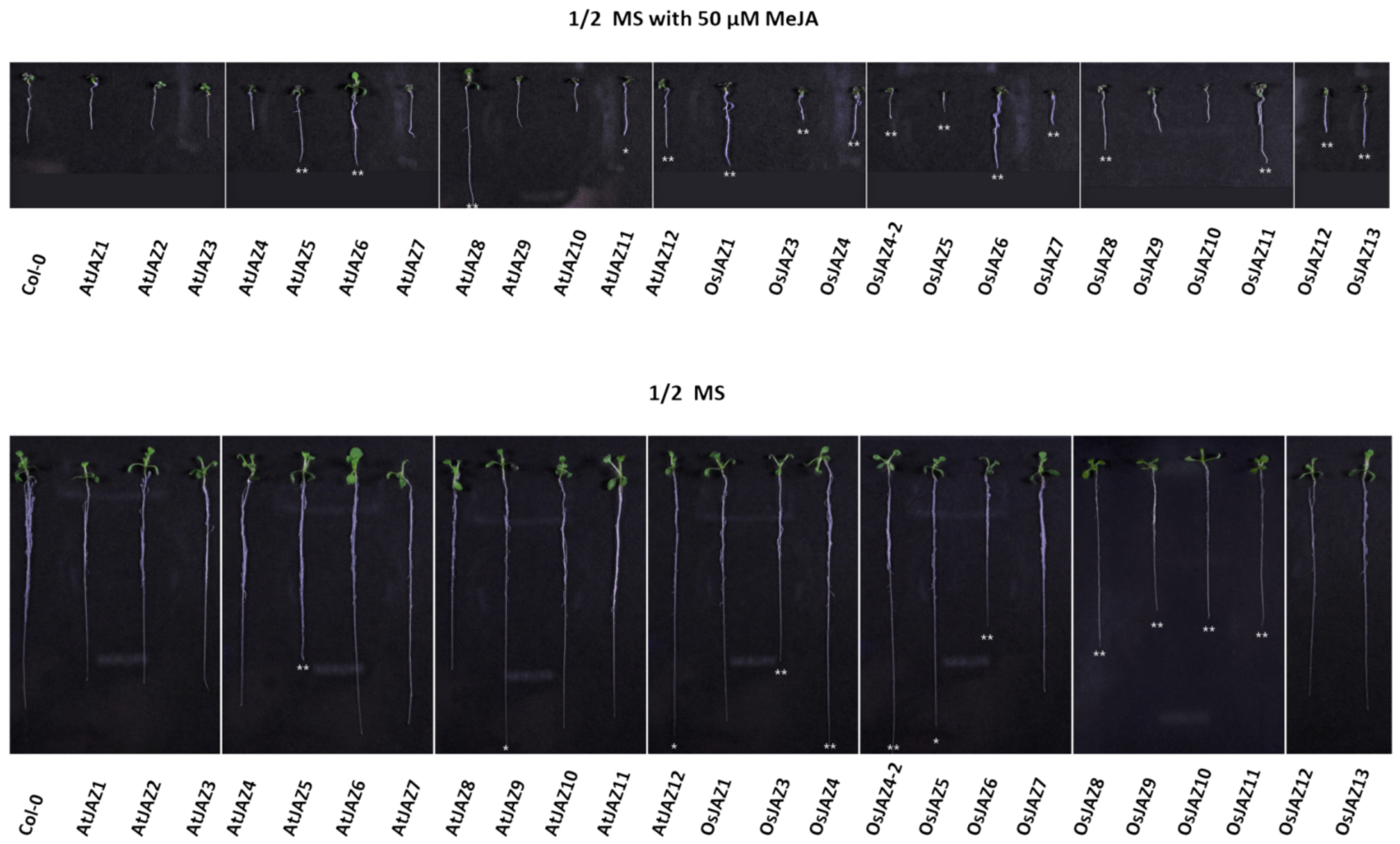

Figure S5. Functions of JAZs in root length. The images were taken when *Arabidopsis thaliana* was vertical grown for 10 days on 1/2-strength MS media or 1/2-strength MS media with 50 μM MeJA. The *Arabidopsis thaliana* plants were grown in a growth chamber at 22 °C under 16 h of light (100 $\mu E \cdot m^{-2} \cdot s^{-1}$) and 8 h of darkness.

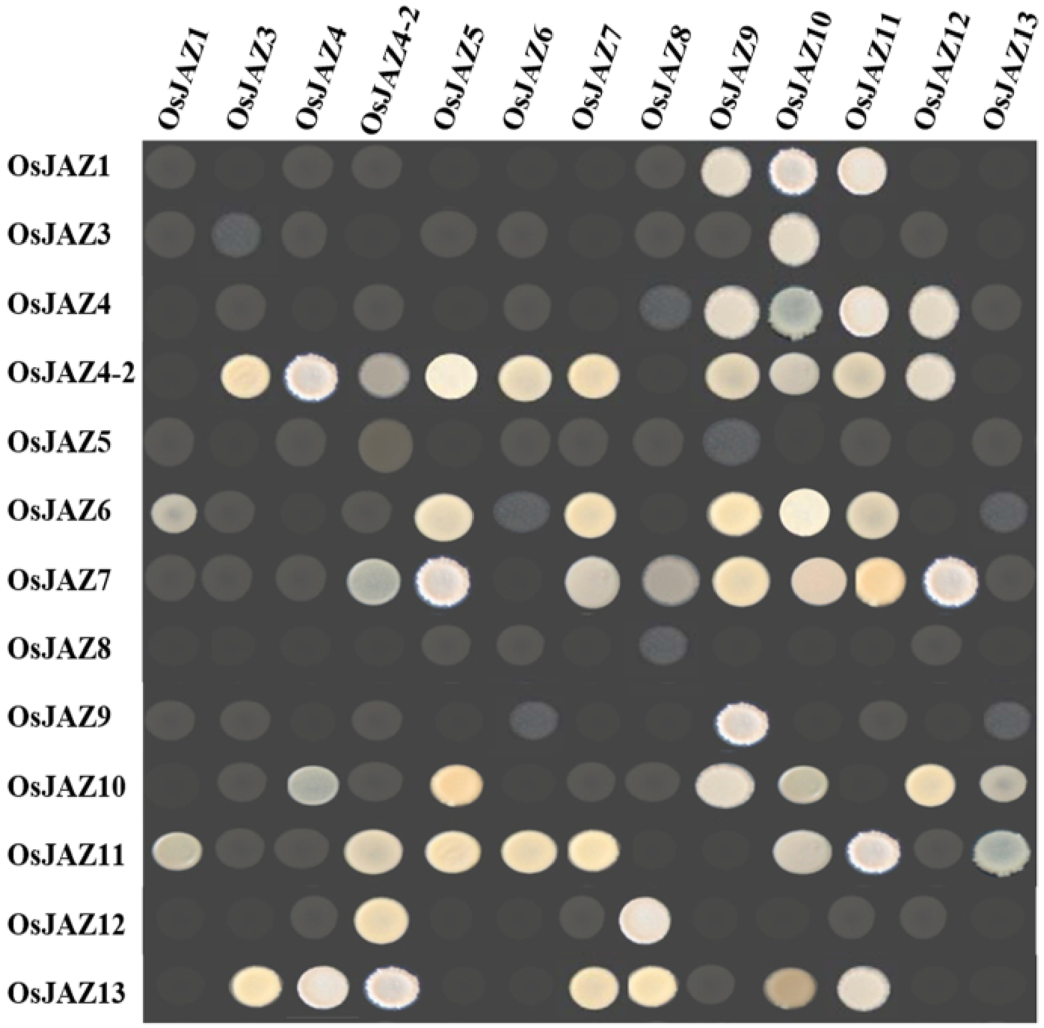

Figure S6. Yeast two-hybrid (Y2H) analysis. Y2H analysis of 13 full-length rice JAZs in both orientations.
